# The prognostic significance of stroke volume index in low gradient severe aortic stenosis: from the national echo database of Australia

**DOI:** 10.1007/s10554-023-02886-y

**Published:** 2023-06-10

**Authors:** Afik D. Snir, Martin K. Ng, Geoff Strange, David Playford, Simon Stewart, David S. Celermajer

**Affiliations:** 1 Faculty of Medicine and Health, University of Sydney, Sydney, NSW Australia; 2 Department of Cardiology, Royal Prince Alfred Hospital, NSW Camperdown, Australia; 3 University of Notre Dame, Fremantle, WA Australia; 4 Heart Research Institute, Newtown, NSW Australia; 5 University of Glasgow, Glasgow, Scotland

**Keywords:** Aortic stenosis, Low-flow, Low-gradient, Stroke volume index

## Abstract

Approximately 50% of patients with severe aortic stenosis (AS) in clinical practice present with ‘low-gradient’ haemodynamics. Stroke Volume Index (SVI) is a measure of left ventricular output, with ‘normal-flow’ considered as  > 35 ml/m^2^. The association between SVI and prognosis in severe low-gradient AS (LGAS) in currently not well-understood. We analysed the National Echo Database of Australia (NEDA) and identified 109,990 patients with sufficiently comprehensive echocardiographic data, linked to survival information. We identified 1,699 with severe LGAS and preserved ejection fraction (EF) (≥ 50%) and 774 with severe LGAS and reduced EF. One- and three-year survival in each subgroup were assessed (follow-up of 74 ± 43 months), according to SVI thresholds. In patients with preserved EF the mortality “threshold” was at SVI < 30 ml/m^2^; 1- and 3-year survival was worse for those with SVI < 30 ml/m^2^ relative to those with SVI > 35 ml/m^2^ (HR 1.80, 95% CI 1.32–2.47 and HR 1.38, 95% CI 1.12–1.70), while survival was similar between those with SVI 30–35 ml/m^2^ and SVI > 35 ml/m^2^. In patients with reduced EF the mortality “threshold” was 35 ml/m^2^; 1- and 3-year survival was worse for both those with SVI < 30 ml/m^2^ and 30–35 ml/m^2^ relative to those with SVI > 35 ml/m^2^ (HR 1.98, 95% CI 1.27–3.09 and HR 1.41, 95% CI 1.05–1.93 for SVI < 30 ml/m^2^ and HR 2.02, 95% CI 1.23–3.31 and HR 1.56, 95% CI 1.10–2.21 for SVI 30–35 ml/m^2^). The SVI prognostic threshold for medium-term mortality in severe LGAS patients is different for those with preserved LVEF (< 30 ml/m^2^) compared to those with reduced LVEF (< 35 ml/m^2^).

**Supplementary Information:**

The online version contains supplementary material available at 10.1007/s10554-023-02886-y.

## Introduction

Approximately 50% of patients with echocardiographic evidence of severe aortic stenosis (AS) in routine clinical practice present with ‘low-gradient’ haemodynamics [1]. This most commonly occurs due to a state of reduced transvalvular flow—commonly known as “low-flow, low-gradient” (LFLG) severe AS [2]. In comparison to the well-characterised group with high-gradient severe AS, LFLG severe AS patients are relatively under-researched and thus present clinicians with greater diagnostic and treatment challenges [3]. Being a heterogeneous group, LFLG severe AS patients are further subdivided into those with ‘preserved’ Left Ventricular Ejection Fraction (LVEF) (≥ 50%) and those with reduced LVEF (< 50%) [4].

Low-flow is conventionally defined as a left ventricular stroke volume index (SVI) of  ≤ 35 ml/m^2^ [5]. Currently, there is limited and conflicting evidence regarding the prognostic SVI threshold in patients with low-gradient severe AS and preserved LVEF [6, 7], with more recent data suggestive a lower SVI threshold (30 ml/m^2^) may be more informative [6]. Moreover, recent evidence [8] also suggested a different SVI prognostic threshold between man and women undergoing aortic valve replacement (AVR). Additionally, to our knowledge, the prognostic implications of different SVI levels have not yet been investigated at all, in those with low-gradient severe AS and reduced LVEF.

In this context, the aim of the current study was to evaluate the prognostic association of different SVI thresholds in patients with severe low-gradient AS and (i) preserved or (ii) reduced LVEF. These entities are often termed “classical” and “paradoxical” LFLG AS, respectively [1]. We analysed data from the National Echo Database of Australia (NEDA)—a large Australia-wide registry containing data from over one million echocardiograms, from more than 600,000 adults being routinely investigated for heart disease. This unique resource has already generated clinically important insights into the prevalence and outcomes of AS [9, 10] and LFLG AS specifically [1, 11].

## Methods

### NEDA design and data

The purpose and overall design of the large, multicentre clinical registry NEDA have been previously described [12]. In brief, NEDA is an ongoing observational registry containing detailed echocardiographic and basic demographic data of adults from over 25 participating centres around Australia. At the time of the most recent study census, the NEDA database contained more than one million echo reports from more than 600,000 individual patients. Survival status (including date of death) for each patient in the database was obtained at this study census date (May 2019) using enhanced probability matching linkage with the well-validated and comprehensive Australian National Death Index [13]. Data from this cohort have been analysed and published previously [1], to characterize the prevalence and outcomes of the different subclasses of severe AS. The current study utilises retrospective data analysis from NEDA to focus specifically on the prognostic significance of SVI in severe low-gradient AS.

### Study cohort

From the entire NEDA database at time of study census (May 2019), only patients  ≥ 18 years with echocardiographic investigations performed since the year 2000 and reporting all the parameters necessary for the accurate diagnosis of severe low-gradient AS were considered for this analysis. Hence, we included only echocardiographic investigations with available Aortic Valve (AV) Area, AV peak velocity, AV mean gradient, SVI and LVEF data. Additionally, for patients with multiple available serial echocardiographic studies in the database, only the first chronological investigation was included.

This resulted in an initial assessment of 109,990 patients to specifically identify those with echocardiographic evidence of severe native AV low-gradient stenosis (see Supplemental Data, Figure S1). Patients were considered to have severe low-gradient AS using criteria based on current expert recommendations [2]. Severe low-gradient AS with preserved ejection fraction (EF) was defined as AVA ≤ 1 cm^2^ with AV mean gradient < 40 mmHg, AV peak velocity < 4 m/s and LVEF ≥ 50%. Severe low-gradient AS with reduced EF was defined as AVA ≤ 1 cm^2^ with AV mean gradient < 40 mmHg, AV peak velocity < 4 m/s and LVEF < 50%. The final analysed cohort included two groups: 1,699 patients (mean age 75 ± 14 years, 63% female) with echocardiographic parameters consistent with severe low-gradient AS and preserved EF and 774 patients (mean age 77 ± 12 years, 36% female) with parameters consistent with severe low-gradient AS and reduced EF.

In each severe low-gradient AS group, subjects were divided into four subgroups according to previously defined SVI thresholds [14]: SVI > 35 ml/m^2^ (normal flow), SVI 30–35 ml/m^2^ (mild low-flow), SVI 25–30 ml/m^2^ (moderate low-flow) and SVI < 25 ml/m^2^ (severe low-flow). Additionally, for a supplemental analysis (see Statistical Analysis section below), the “normal flow” subgroup was further divided into low-normal flow (SVI 35–40 ml/m^2^) and high-normal flow (SVI > 40 ml/m^2^). AVA was calculated from the continuity equation using either the Velocity Time Integral (VTI) and/or peak velocity ratio [15], with the minimum value obtained for each patient used for classifying the type of severe AS. Stroke volume was calculated by the Doppler method in accordance with guideline recommendation [15], by multiplying the measured LV outflow tract (LVOT) area with the LVOT VTI, and indexed to patient body surface area (BSA). LVEF was obtained by following hierarchal methods: volumetric apical biplane (Simpson’s), volumetric apical four-chamber, volumetric apical two-chamber, or finally by the Teichholz formula. Cardiac Damage Staging [16] for each patient was calculated as recently described elsewhere using available echo parameters [11]. The presence of Atrial Fibrillation rhythm at time of echocardiography was inferred from the E and A velocity profile measured at the mitral valve inflow using pulse wave Doppler.

### Survival outcomes

Survival at 1- and 3-years was assessed according to SVI subgroup separately in subjects with severe low-gradient AS and preserved EF and in subjects with severe low-gradient AS and reduced EF. The follow-up period for each patient was from the time of diagnostic transthoracic echocardiogram to time of study census (May 2019, as above). Mean follow-up was 76 (± 41) months in the severe low-gradient AS with preserved EF group and 81 (± 42) months in the severe low-gradient AS with reduced EF group.

### Aortic valve replacement (AVR) status

As previously reported [11], AVR status was identified using text recognition software of the free text and conclusions of each echo report in the database, rather than with direct linkage to national surgical or interventional databases. Therefore, patients were only recorded to have undergone AVR during follow-up, either surgically or with a TAVI procedure, if any of their subsequent available echocardiograms in the database reported evidence of a replaced or implanted AV.

### Statistical analysis

Continuous variables are presented as mean (± standard deviation) and categorical variables are presented as percent (count). For each low-gradient severe AS group (preserved or reduced EF), comparisons between SVI subgroups were assessed by student’s t-test, chi-square test or ANOVA with Bonferroni correction as appropriate. One- and three-year survival curves for each severe low-gradient AS group were plotted using the Kaplan–Meier method (using separate analyses), with patients censored at last known survival status (i.e. at study census, May 2019). Additionally, survival was further assessed with Cox proportional hazards multivariable regression models including SVI subgroup (as defined above), age, sex, Body Mass Index (BMI), indexed AVA (AVAi) and Cardiac Damage Stage as co-variates.

We performed sensitivity survival analyses for both severe low-gradient AS groups (see Supplementary data) to investigate the specific effect of patient sex on outcomes. Additionally, we repeated the original survival analyses for patients with preserved EF while dividing them into the five prospectively defined SVI subgroups (i.e. SVI < 25 ml/m^2^, SVI 25–30 ml/m^2^, SVI 30–35 ml/m^2^, SVI 35–40 ml/m^2^ and SVI > 40 ml/m^2^). Moreover, we repeated the multivariable regression analysis for patients with reduced EF to also specifically include LVEF% as an additional co-variate in the model.

Statistical calculations were performed using SPSS software (IBM, Armonk, New York) and significance was inferred at a 2-sided p value of < 0.05.

## Results

The results of (A) patients with severe low-gradient AS and preserved EF (i.e. paradoxical LFLG severe AS) and (B) those with severe low-gradient AS and reduced EF (i.e. classical LFLG severe AS) are presented separately below.

### Severe low-gradient AS with preserved EF patients

In these 1699 adults, age was 75 ± 14 years and 63% were female (Table 1). The mean and median SVI were 33.4 (± 10.6) and 33.0 (IQR 25.7–40.8) ml/m^2^, respectively. Overall, the average BMI was 27.5 (± 6.4) kg/m^2^, AV mean gradient was 22.3 (± 9.8) mmHg, indexed AVA was 0.51 (± 0.14) cm^2^/m^2^ and LVEF was 63.5% (± 7.9%). Lower SVI was significantly associated with higher BMI (p < 0.001), smaller LVOT diameter (p < 0.001), smaller AVA indexed (p < 0.001), lower AV mean gradient and peak velocity (p < 0.001), more advanced Cardiac Damage Staging (stage 3/4, p < 0.05) and lower recorded rate of AVR during follow-up (p < 0.05).

**Table 1:** Baseline Group Characteristics According to SVI subgroup in patients with low-gradient severe AS and preserved LVEF (≥ 50%)

Variable	SVI < 25 ml/m^2^ (n = 392)	SVI 25-30 ml/m^2^ (n = 261)	SVI 30-35 ml/m^2^ (n = 306)	SVI > 35 ml/m^2^ (n = 740)
Age (years)	73.2 (± 14.7)**	75.3 (± 13.9)	74.7 (± 14.4)	76.8 (± 12.7)
Female sex	69.6% (273)*	58.6% (153)*	57.5% (176)*	63.5% (470)
BMI (kg/m^2^)	29.2 (± 7.4)**	28.1 (± 6.3)**	28.2 (± 6.6)**	26.3 (± 5.4)
BSA (m^2^)	1.86 (± 0.27)**	1.84 (± 0.28)**	1.83 (± 0.27)**	1.74 (± 0.22)
LVOT diameter (cm)	1.73 (± 0.27)**	1.89 (± 0.26)**	1.95 (± 0.24)**	2.07 (± 0.21)
AVA (VTI) (cm^2^)	0.83 (± 0.26)**	0.88 (± 0.28)*	0.89 (± 0.24)*	0.94 (± 0.16)
Index AVA (VTI) (cm^2^/m^2^)	0.45 (± 0.14)**	0.49 (± 0.16)**	0.49 (± 0.14)**	0.55 (± 0.12)
AV mean gradient (mmHg)	12.9 (± 8.6)**	19.3 (± 8.9)**	23.2 (± 8.2)**	27.5 (± 7.1)
AV peak velocity (m/s)	2.3 (± 0.7)**	2.8 (± 0.6)**	3.1 (± 0.6)**	3.4 (± 0.4)
LVEF (%)	62.7 (± 8.2)	62.7 (± 7.4)	63.9 (± 7.9)	63.8 (± 7.6)
LVEDD (cm)	4.3 (± 0.6)	4.2 (± 0.7)	4.3 (± 0.6)	4.3 (± 0.6)
LVESD (cm)	2.8 (± 0.6)	2.7 (± 0.6)	2.7 (± 0.5)	2.7 (± 0.6)
LV mass indexed (g/m^2^)	87.7 (± 27.8)**	92.7 (± 26.9)	95.2 (± 25.8)	98.0 (± 27.7)
E/e' ratio	14.9 (± 6.8)**	17.2 (± 8.2)	17.7 (± 7.2)	18.3 (± 8.1)
LA volume indexed (ml/m^2^)	36.1 (± 19.3)**	43.8 (± 23.7)	40.3 (± 16.9)	44.4 (± 18.8)
RVSP (mmHg)	42.2 (± 16.5)	42.0 (± 13.3)	41.2 (± 13.0)	40.5 (± 13.1)
Moderate-severe MR	11.0% (43)	12.3% (32)	11.8% (36)	15.8% (117)
Moderate-severe TR	21.2% (83)**	17.2% (45)	15.0% (46)	11.6% (86)
Atrial fibrillation	26.0% (102)**	23.0% (60)*	21.2% (65)*	13.8% (102)
Paced rhythm	4.3% (17)	3.4% (9)	2.6% (8)	2.4% (18)
Cardiac damage stage				
Stage 0	37% (145)*	33.3% (87)	31.4% (96)	28.9% (214)
Stage 1	12% (47)	18.0% (47)	21.9% (67)	15.8% (117)
Stage 2	26.5% (104)**	25.3% (66)*	28.1% (86)*	40.3% (298)
Stage 3/4	24.5% (96)*	23.4% (61)*	18.6% (57)	15.0% (111)
AVR during follow-up†	7.1%**	10.3%**	17.6%*	24.9%
Mean follow-up (months)	60.4 (± 33.8)**	66.2 (± 40.5)**	74.3 (± 42.8)	80.7 (± 45.9)
1-year mortality	16.8% (66)	18.0% (47)	13.1% (40)	10.4% (77)
3-year mortality	31.9% (125)	30.7% (80)	29.1% (89)	28.2% (209)

*p < 0.05 comparing to SVI  > 35 ml/m^2^ group, **p ≤ 0.001 comparing to SVI > 35 ml/m^2^ group, †AVR recorded only if noted on subsequent TTE during follow-up (rather than from clinical records)

BMI; Body Mass Index, BSA; Body Surface Area, LVOT; Left Ventricle Outflow Tract, AVA; Aortic Valve Area, AV; Aortic Valve, LVEF; Left Ventricle Ejection Fraction, LVEDD; Left Ventricle End Diastolic Diameter, LVESD; Left Ventricle End Systolic Diameter, LV; Left Ventricle, LA; Left Atrium, RVSP; Right Ventricle Systolic Pressure, MR; Mitral Regurgitation, TR; Tricuspid Regurgitation, AVR; Aortic Valve Replacement

**Fig. 1:**
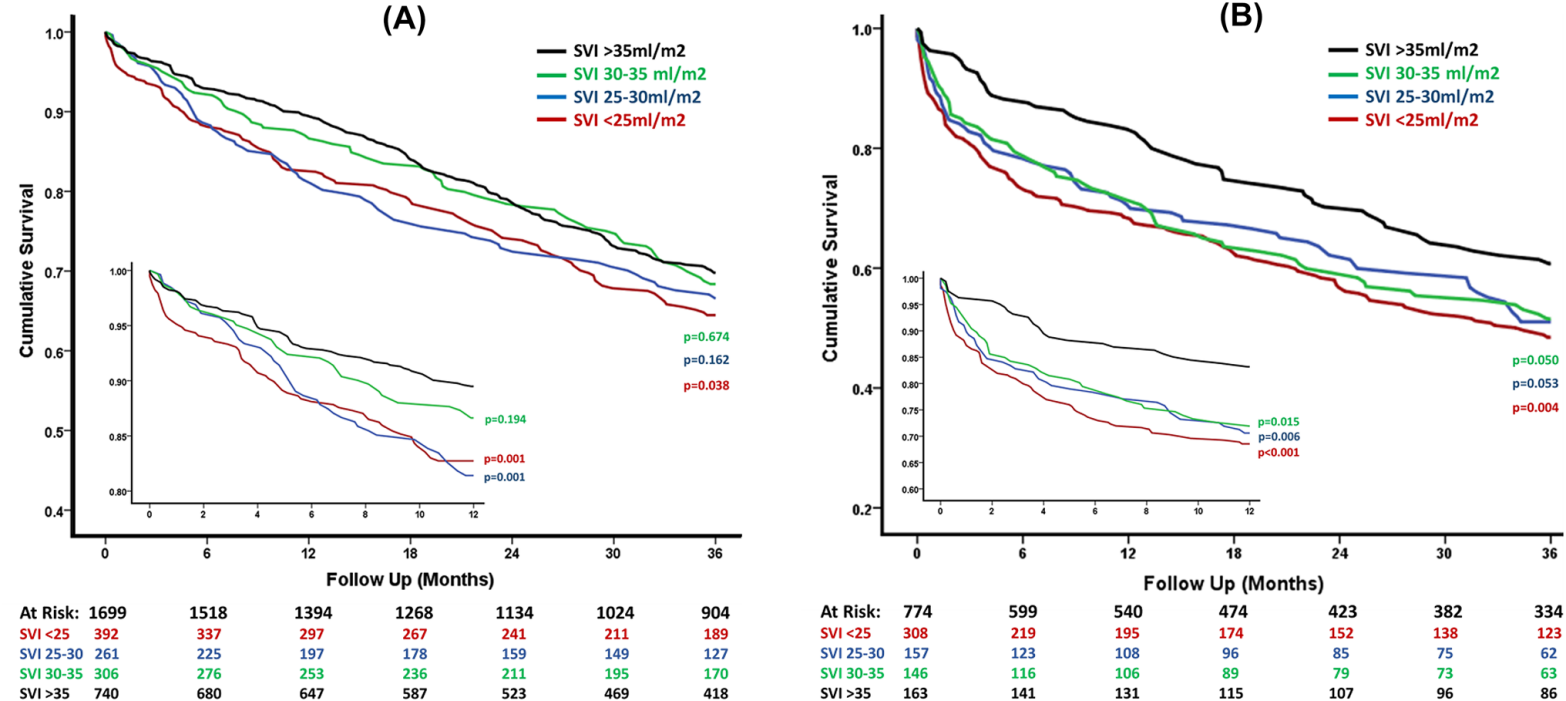
Showing Kaplan–Meier curves for 3-year survival (main figure) and 1-year survival (small internal figure) according to SVI subgroup, in patients with **A** severe low-gradient AS and preserved EF (≥ 50%) and **B** severe low-gradient AS and reduced EF (< 50%)

**Fig. 2:**
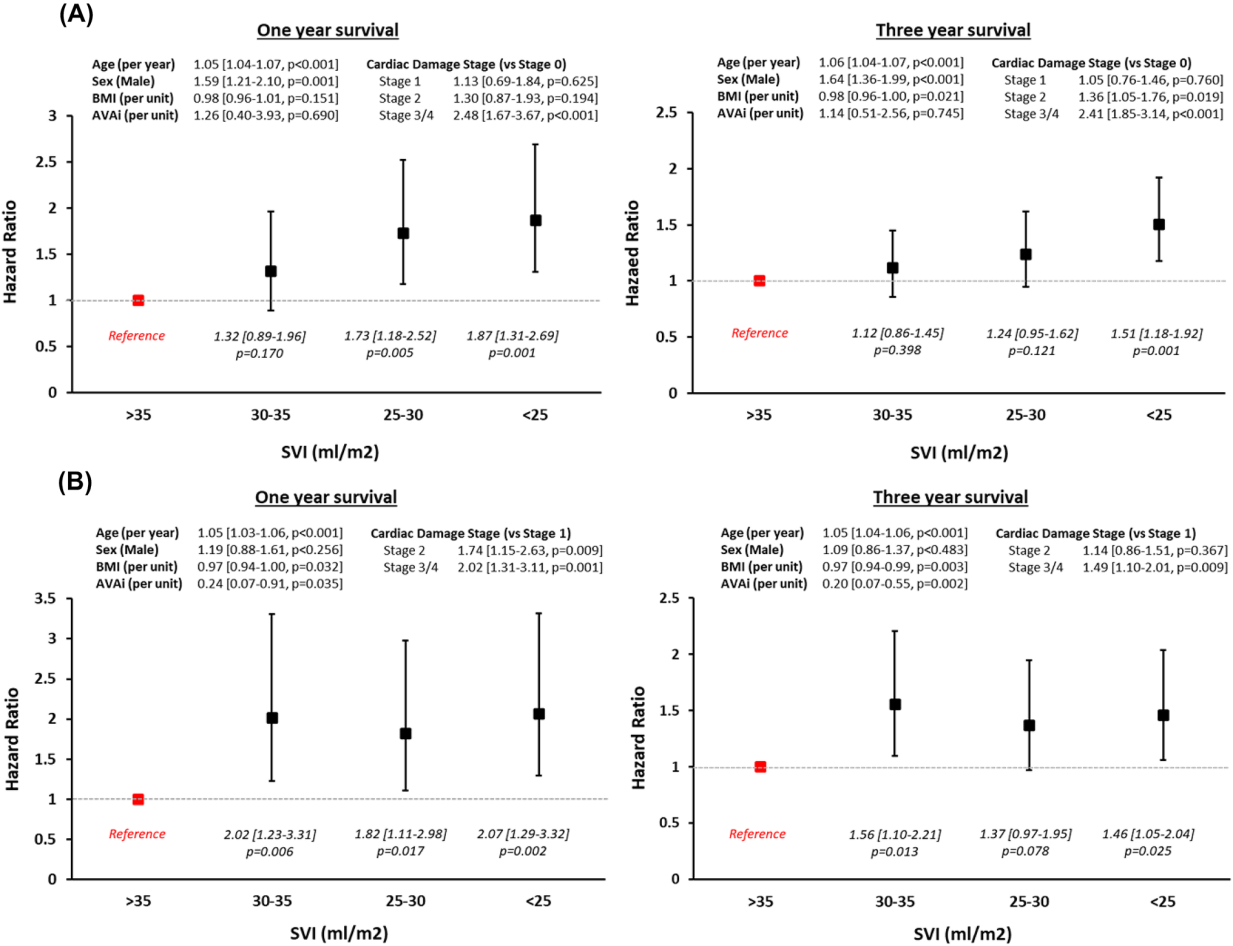
Results of Cox multivariable regression analyses. Showing adjusted HR [± 95% CI] according to SVI subgroup (with reference to patients with SVI > 35 ml/m^2^) for both 1- and 3-year survival in **A** patients with severe low-gradient AS and preserved EF (≥ 50%) and **B** patients with severe low-gradient AS and reduced EF (< 50%)

Figure 1 shows the Kaplan–Meier survival curves according to SVI subgroup, for these patients. Overall 1- and 3-year survival was 90% and 72% for patients with SVI > 35 ml/m^2^, 87% and 71% for patients with SVI 30–35 ml/m^2^, 82% and 69% for patients with SVI 25–30 ml/m^2^ and 83% and 68% for patients with SVI < 25 ml/m^2^. Following adjustment with multivariate analysis (Fig. 2), compared to those with SVI > 35 ml/m^2^, 1-year survival was significantly lower only for patients with SVI < 25 ml/m2 (HR 1.87, 95% CI 1.31–2.69) and SVI 25–30 ml/m^2^ (HR 1.72, 95% CI 1.18–2.52) while 3-year survival was significantly lower only in those with SVI < 25 ml/m^2^ (HR 1.51, 95% CI 1.18–1.92). When considering all patients with SVI < 30 ml/m^2^ as one group, both 1- and 3-year survival was independently worse than those with SVI > 35 ml/m^2^ (HR 1.80, 95% CI 1.32–2.47 and HR 1.38, 95% CI 1.12–1.70, respectively). There was no statistical difference in either adjusted 1- or 3-year survival between those with SVI 30-35 ml/m^2^ and those with SVI > 35 ml/m^2^ (HR 1.32, 95% CI 0.89–1.96 and HR 1.12, 95% CI 0.85–1.45, respectively).

The additional supplementary analyses revealed no prognostic difference between those with SVI > 40 ml/m^2^ and SVI 35–40 ml/m^2^ (Supplementary data, Figure S2 and Table S1). When considering only female patients (n = 1072), only those with SVI < 25 ml/m^2^ had significantly worse 1-year survival (p = 0.015) compared with the SVI > 35 ml/m^2^ subgroup, while there was no significant survival difference at 3-years between all SVI subgroups (Supplementary data, Figure S3 and Table S2). When considering only male patients (n = 627), survival at 1- and 3-years (Supplementary data, Figure S4 and Table S3) was significantly lower in both the SVI 25-30 ml/m^2^ (p = 0.019 and p = 0.023, respectively) and SVI < 25 ml/m^2^ (p = 0.026 and p = 0.002, respectively) subgroups, compared to those with SVI > 35 ml/m^2^.

### Severe low-gradient AS with reduced EF patients

In these 774 adults, age was 77 ± 12 years and 36% were female (Table 2). The mean and median SVI were 27.6 (± 9.6) and 27.6 (IQR 20.8–34.0) ml/m^2^, respectively. Overall, the average BMI was 26.5 (± 5.5) kg/m^2^, AV mean gradient was 21.1 (± 9.8) mmHg, indexed AVA was 0.47 (± 0.15) cm^2^/m^2^ and LVEF was 34.5% (± 10.3%). Lower SVI was significantly associated with smaller LVOT diameter and AVA indexed (p < 0.05), lower AV mean gradient and peak velocity (p < 0.05), lower LVEF (p < 0.001) and lower recorded rate of AVR during follow-up (p < 0.05).

**Table 2:** Baseline Group Characteristics According to SVI subgroup in patients with low-gradient severe AS and reduced LVEF (< 50%)

Variable	SVI < 25 ml/m^2^ (n = 308)	SVI 25-30 ml/m^2^ (n = 157)	SVI 30-35 ml/m^2^ (n = 146)	SVI > 35 ml/m^2^ (n = 163)
Age (years)	75.2 (± 12.9)**	77.0 (± 11.8)	77.3 (± 11.0)	79.3 (± 8.5)
Female sex	36.0% (111)	35.7% (56)	37.7% (55)	35.6% (58)
BMI (kg/m^2^)	27.3 (± 5.6)*	26.5 (± 6.0)	26.6 (± 5.5)	25.7 (± 4.7)
BSA (m^2^)	1.90 (± 0.25)**	1.86 (± 0.27)	1.84 (± 0.23)	1.79 (± 0.23)
LVOT diameter (cm)	1.99 (± 0.29)**	2.13 (± 0.24)*	2.19 (± 0.22)	2.22 (± 0.22)
AVA (VTI) (cm^2^)	0.80 (± 0.31)**	0.86 (± 0.33)	0.86 (± 0.13)	0.93 (± 0.15)
Index AVA (VTI) (cm^2^/m^2^)	0.43 (± 0.16)**	0.47 (± 0.18)*	0.47 (± 0.10)*	0.53 (± 0.10)
AV mean gradient (mmHg)	15.3 (± 9.4)**	21.8 (± 8.8)**	24.6 (± 7.3)*	27.8 (± 7.3)
AV peak velocity (m/s)	2.5 (± 0.8)**	3.0 (± 0.6)**	3.2 (± 0.4)*	3.4 (± 0.4)
LVEF (%)	31.2 (± 11.1)**	34.2 (± 9.5)**	36.7 (± 9.2)	39.1 (± 7.8)
LVEDD (cm)	5.3 (± 0.9)	5.2 (± 0.9)	5.0 (± 0.7)	5.0 (± 0.8)
LVESD (cm)	4.4 (± 1.1)*	4.2 (± 1.0)	3.9 (± 0.8)	4.0 (± 0.8)
LV mass indexed (g/m^2^)	118.6 (± 34.9)	125.6 (± 34.7)	126.2 (± 36.0)	125.9 (± 32.5)
E/e' ratio	22.6 (± 11.1)	20.2 (± 8.4)	20.9 (± 8.3)	22.3 (± 9.1)
LA volume indexed (ml/m^2^)	53.8 (± 18.4)	55.0 (± 15.9)	54.5 (± 19.0)	55.0 (± 21.5)
RVSP (mmHg)	46.0 (± 13.3)	47.2 (± 12.0)	44.6 (± 12.5)	44.4 (± 13.3)
Moderate-severe MR	32.8% (101)	33.8% (53)	31.5% (46)	30.7% (50)
Moderate-severe TR	35.1% (108)**	23.6% (37)	11.0% (16)	11.7% (19)
Atrial fibrillation	39.0% (120)**	29.9% (47)	21.9% (32)	22.7% (37)
Paced rhythm	7.1% (22)	3.8% (6)	4.8% (7)	1.8% (3)
Cardiac damage stage				
Stage 1	19.2% (59)	26.8% (42)	32.2% (47)	25.2% (41)
Stage 2	38.0% (117)*	42.0% (66)	50.0% (73)	54.0% (88)
Stage 3/4	42.8% (132)**	31.2% (49)	17.8% (26)	20.8% (34)
AVR during follow-up†	12.0% (37)**	15.3% (24)*	28.1% (41)	28.2% (46)
Mean follow-up (months)	76.5 (± 42.0)	75.6 (± 46.2)	75.3 (± 41.6)	86.9 (± 45.2)
1-year mortality	30.8% (95)	29.3% (46)	28.1% (41)	16.6% (27)
3-year mortality	48.4% (149)	45.9% (72)	47.3% (69)	37.4% (61)

*p < 0.05 comparing to SVI  > 35 ml/m^2^ group, **p ≤ 0.001 comparing to SVI  > 35 ml/m^2^ group, †AVR recorded only if noted on subsequent TTE during follow-up (rather than from clinical records)

BMI; Body Mass Index, BSA; Body Surface Area, LVOT; Left Ventricle Outflow Tract, AVA; Aortic Valve Area, AV; Aortic Valve, LVEF; Left Ventricle Ejection Fraction, LVEDD; Left Ventricle End Diastolic Diameter, LVESD; Left Ventricle End Systolic Diameter, LV; Left Ventricle, LA; Left Atrium, RVSP; Right Ventricle Systolic Pressure, MR; Mitral Regurgitation, TR; Tricuspid Regurgitation, AVR; Aortic Valve Replacement

Figure 1 shows the Kaplan–Meier survival curves according to SVI group. Overall 1- and 3-year survival was 83% and 63% for patients with SVI > 35 ml/m^2^, 72% and 53% for patients with SVI 30–35 ml/m^2^, 71% and 54% for patients with SVI 25-30 ml/m^2^ and 69% and 52% for patients with SVI < 25 ml/m^2^. Following adjustment with multivariate analysis (Fig. 2), compared to patients with SVI > 35 ml/m^2^, 1-year survival was significantly worse in all lower SVI subgroups (HR 2.02, 95% CI 1.23–3.31 for SVI 30–35 ml/m^2^, HR 1.82, 95% CI 1.11–2.98 for SVI 25–30 ml/m^2^ and HR 2.07, 95% CI 1.29–3.32 for SVI < 25 ml/m^2^). Similarly, 3-year survival was significantly lower for those with SVI 30–35 ml/m^2^ (HR 1.56, 95% CI 1.10–2.21) and with SVI < 25 ml/m^2^ (HR 1.46, 95% CI 1.05–2.04), but not statistically significant for those with SVI 25–30 ml/m^2^ (HR 1.37, 95% CI 0.97–1.95). When combining all patients with SVI ≤ 35 ml/m^2^ together, both 1- and 3-year survival were independently worse than in patients with SVI > 35 ml/m^2^ (HR 1.99, 95% CI 1.30–3.07 and HR 1.46, 95% CI 1.09–1.96, respectively).

This observed prognostic SVI threshold (i.e. < 35 ml/m^2^) remained significant following further adjustment for LVEF as an additional variable on supplemental multivariate analyses (HR 1.77, 95% CI 1.15–2.74 for 1-year survival and HR 1.38, 95% CI 1.03–1.86 for 3-year survival). There were no significant differences in the survival trends between male and female patients.

## Discussion

The results from this large study of nearly 2,500 patients with severe low-gradient AS on transthoracic echocardiogram, identified from routine clinical practice, contribute to our understanding of the prognostic significance of SVI in this less well understood subset of patients with severe AS. Specifically, to our knowledge, this represents the first investigation into the association between SVI and survival in patients with low-gradient severe AS and reduced LVEF (< 50%). We found that 1- to 3-year survival was reduced below a SVI threshold of < 30 ml/m^2^ in those with preserved LVEF and below a SVI threshold of < 35 ml/m^2^ in those with reduced LVEF.

SVI is a measurement of left ventricular forward output; SVI < 35 ml/m^2^ is accepted [2] to represent a state of reduced LV output, with SVI 25–30 ml/m^2^ and SVI < 25 ml/m^2^ representing moderately and severely reduced output, respectively [14]. In previous smaller studies, SVI ≤ 35 ml/m^2^ has generally been associated with worse outcomes in adults with severe AS, either in those undergoing AVR or conservative management [7, 17-20]. By contrast, Rusinaru and colleagues [6] examined 395 patients with low-gradient severe AS and preserved LVEF (≥ 50%) and found that there was no survival difference between having a SVI > 35 ml/m^2^ or SVI 30-35 ml/m^2^, including on multivariate analysis. It is also noteworthy, regarding SVI “cut-points”, that nearly 40% of adults with a “normal echocardiogram” have a SVI ≤ 35 ml/m^2^ [6].

Similar to the findings of Rusinaru, our analysis of 1,699 patients with severe low-gradient AS and preserved LVEF (≥ 50%) also showed no difference in medium-term survival between those with SVI > 35 ml/m^2^ or with SVI 30–35 ml/m^2^. Compared to those with ‘normal’ flow, the relative mortality risk on multivariate analysis for patients with SVI < 30 ml/m^2^ was 80% higher at 1-year and 38% higher at 3-years. Unlike the previous analysis by Eleid et al. [7], we did not find a prognostic benefit in our cohort of having ‘high-normal’ flow haemodynamics (i.e. SVI > 40 ml/m^2^) over the conventional SVI cut-off of 35 ml/m^2^.

In contrast, results from the 774 patients we studied with severe low-gradient AS and reduced LVEF (< 50%) showed a clear prognostic threshold at 35 ml/m^2^. Those with SVI < 35 ml/m^2^ had an elevated relative mortality risk on multivariate analysis by 80–100% at 1-year and by 40–50% at 3-years, compared to those with ‘normal’ flow. This effect remained very similar following additional adjustment for calculated LVEF% on multivariate analysis, beyond the original adjustment for the extent of extra aortic valve cardiac disease using the established Cardiac Damage Staging Classification [11, 16, 17].

Interestingly, our supplementary analyses showed a substantially less marked association between SVI and survival for female compared to male patients in the preserved EF group. One possible explanation is the higher rates of AVR recorded in the male patients (24% vs. 13%, overall, p < 0.001), albeit this measurement is not very reliable and likely represents an underestimation of the true AVR rate (as further discussed in the limitations section below). Alternatively, the lower indexed AVA measured in male patients (mean of 0.48 vs. 0.52cm^2^/m^2^ in females, p < 0.001) could suggest a greater likelihood of truly severe AS in this subpopulation. This raises the important consideration that a proportion of patients with low AV gradients and preserved EF (particularly in females and/or those with only mild-moderate reduced SVI) may in-fact have had “pseudo-severe” AS due to underestimation of LVOT diameter measurement, which will affect both the AVA and SVI calculations [18]. Indeed, this argument is supported by the fact that, on multivariate analysis, indexed AVA (as a continuous variable) was significantly associated with survival in the group with reduced EF but was prognostically insignificant in those with preserved EF. This group of patients with paradoxical LFLG severe AS often present a diagnostic and prognostic challenge and we have previously documented that the main recorded cause of death in such patients is non-cardiac [1].

Considering our findings together with previous data [6] and expert opinion [14], we would argue that a SVI threshold of 30 ml/m^2^ may be more prognostically appropriate in those with severe low-gradient AS with preserved EF, especially in female patients and/or those with BMI > 30 kg/m^2^. SVI levels between 30 and 35 ml/m^2^ are more likely to correspond with ‘normal’ physiology in the presence of relatively small LV size and/or exaggerated BSA in these patient populations. Moreover, we also emphasise the importance of careful assessment of echo-derived haemodynamics measurements as well as utilisation of additional investigations such as CT AV calcium scoring in low-gradient AS with preserved EF patients [2], in order to ensure correct diagnosis and prognostication. Particular attention should be directed to ‘double checking’ the recording and measurement of LVOT diameter and LVOT/AV Doppler tracing, including repeating these following blood-pressure optimisation in those with moderate-severe systemic hypertension.

### Limitations

NEDA incorporates data from a network of over 25 participating centres with both inpatient and outpatient services across all the states of Australia, hence representing ‘real-world’ patient heterogeneity and echocardiographic practice across an advanced healthcare system, servicing an ethnically diverse population. However, there are three main limitations of the database that impact the completeness of this analysis into the association between SVI and survival. The first was the lack of available clinical history such as co-morbid conditions or symptomatic status. Secondly, we were unable to adjust for the expected survival benefit of AVR; the reported incidence of AVR relied on available information from follow-up echocardiograms in the database, therefore undoubtedly underestimating the true incidence of intervention in this cohort (due to early post-operative mortality or loss to follow-up, for example). Thirdly, as our analysed cohort depended on identifying patients with sufficiently available echo data in the database, we cannot confidently exclude potential selection biases. Additionally, there is also the issue of the current lack of expert consensus regarding the most appropriate LVOT diameter measurement strategy (i.e. annular versus sub-annular measurement) [15, 21], which may cause discrepancy in calculated SVI between operators, especially in patients with significant valvular/sub-valvular calcification. This would have been expected to introduce random rather than systematic error, and thus should not have altered the main conclusions from the study.

## Conclusion

We examined the prognostic significance of SVI measured on echocardiography during routine clinical practice in patients with low-gradient severe AS characteristics. There was reduced medium-term survival with SVI < 30 ml/m^2^ in those preserved EF (≥ 50%) and with SVI < 35 ml/m^2^ in those with reduced EF (< 50%). Consistent with previous data, there was no prognostic difference between SVI 30–35 ml/m^2^ and SVI > 35 ml/m^2^ in subjects with low-gradient severe AS and preserved EF. Our results establish the utility of SVI in risk stratification for severe AS patients with low-gradient haemodynamics, adding prognostic value in addition to other established indicators of valvular stenosis and cardiac damage severity.

### Supplementary Information

Below is the link to the electronic supplementary material.

Supplementary file1 (DOCX 1042 KB)
