# Supplementary material for: The prognostic significance of stroke volume index in low gradient severe aortic stenosis: from the national echo database of Australia
Source: Int J Cardiovasc Imaging. 2023 Jun 10;39(9):1719–27. doi: 10.1007/s10554-023-02886-y (PMC10520126; doi:10.1007/s10554-023-02886-y)

**Supplementary Data**

Figure S1 – Study Flowchart


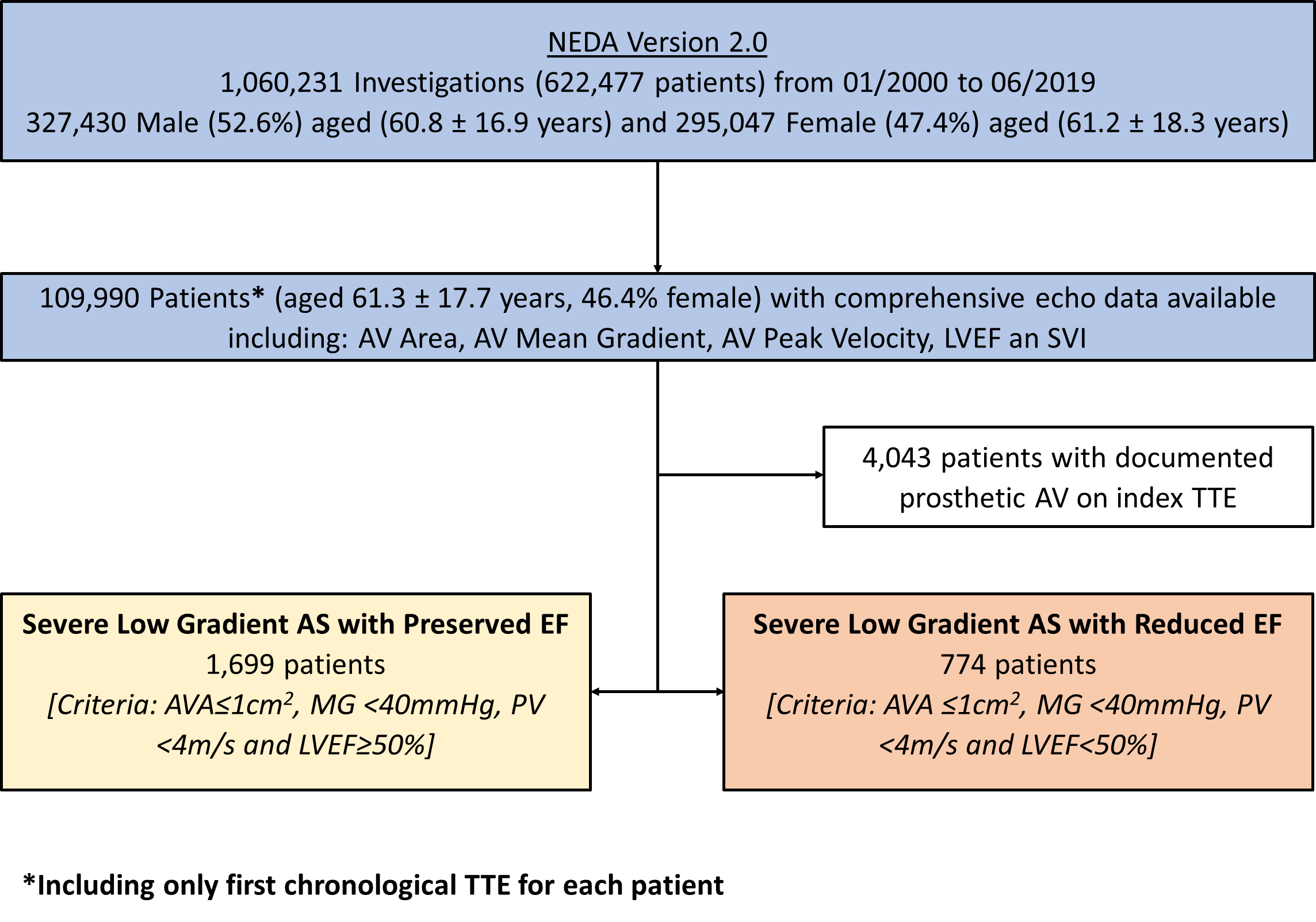


Table S1 – Baseline group characteristics according to SVI subgroup in patients with low-gradient severe AS and preserved LVEF (≥50%)

| **Variable** | **SVI <25ml/m^2^ n=392** | **SVI 25-30ml/m^2^ n=261** | **SVI 30-35ml/m2 n=306** | **SVI 35-40ml/m^2^ n=282** | **SVI >40ml/m^2^ n=458** |
| --- | --- | --- | --- | --- | --- |
| Female Sex | 69.6% (273) | 58.6% (153) | 57.5% (176) | 57.4% (162) | 67.2% (308) |
| Mean BMI (±SD) | 29.2 (±7.4)** | 28.1 (±6.3)** | 28.2 (±6.6)** | 27.0 (±5.9) | 25.8 (±5.0) |
| LVOT Diameter (cm) | 1.73 (±0.27)** | 1.89 (±0.26)** | 1.95 (±0.24)** | 2.04 (±0.23)* | 2.09 (±0.20) |
| AVA (VTI) (cm^2^) | 0.83 (±0.26)** | 0.88 (±0.28)* | 0.89 (±0.24)* | 0.92 (±0.20) | 0.95 (±0.13) |
| Index AVA (VTI) (cm^2^/m^2^) | 0.45 (±0.14)** | 0.49 (±0.16)** | 0.49 (±0.14)** | 0.52 (±0.12)* | 0.56 (±0.14) |
| AV Mean Gradient (mmHg) | 12.9 (±8.6)** | 19.3 (±8.9)** | 23.2 (±8.2)** | 25.3 (±7.3)** | 28.8 (±6.7) |
| AV Peak Velocity (m/s) | 2.3 (±0.7)** | 2.8 (±0.6)** | 3.1 (±0.6)** | 3.3 (±0.4)** | 3.5 (±0.4) |
| LVEF (%) | 62.7 (±8.2) | 62.7 (±7.4) | 63.9 (±7.9) | 63.4 (±7.7) | 64.1 (±7.6) |
| LVEDD (cm) | 4.3 (±0.6) | 4.2 (±0.7) | 4.3 (±0.6) | 4.3 (±0.7) | 4.3 (±0.6) |
| LVESD (cm) | 2.8 (±0.6) | 2.7 (±0.6) | 2.7 (±0.5) | 2.8 (±0.6) | 2.7 (±0.6) |
| RVSP (mmHg) | 42.2 (±16.5) | 42.0 (±13.3) | 41.2 (±13.0) | 39.9 (±13.6) | 40.8 (±12.8) |
| Moderate-Severe MR | 11.0% (43)* | 12.3% (32) | 11.8% (36) | 11.3% (32) | 18.6% (85) |
| Moderate-Severe TR | 21.2% (83)* | 17.2% (45) | 15.0% (46) | 11.3% (32) | 11.8% (54) |
| Cardiac Damage Stage |  |  |  |  |  |
| Stage 0 | 37% (145)* | 33.3% (87) | 31.4% (96) | 30.5% (86) | 27.9% (128) |
| Stage 1 | 12% (47) | 18.0% (47) | 21.9% (67)* | 18.8% (53) | 14.0% (64) |
| Stage 2 | 26.5% (104)** | 25.3% (66)** | 28.1% (86)** | 37.2% (105) | 42.1% (193) |
| Stage 3/4 | 24.5% (96)* | 23.4% (61) | 18.6% (57) | 13.5% (38) | 15.9% (73) |
| AVR during follow-up⸶ | 7.1% (28)** | 10.3% (27)** | 17.6% (54)** | 17.0% (48)** | 29.7% (136) |
| Mean follow-up (months) | 60.4 (±33.8)** | 66.2 (±40.5)** | 74.3 (±42.8)* | 75.4 (±45.5) | 84.0 (±42.8) |

*p value <0.05 comparing to SVI >40ml/m^2^ group, **p value ≤0.001 comparing to SVI >40ml/m^2^ group, ⸶AVR recorded only if noted on subsequent TTE during follow-up (rather than from clinical records).

Figure S2

Showing one- and three-year Kaplan-Meier survival curves in patients with severe low-gradient AS and preserved EF (≥50%) according to SVI subgroup, including results of Cox proportional hazards multivariable regression analyses adjusting for patient age, sex, BMI, indexed AVA and Cardiac Damage Stage.


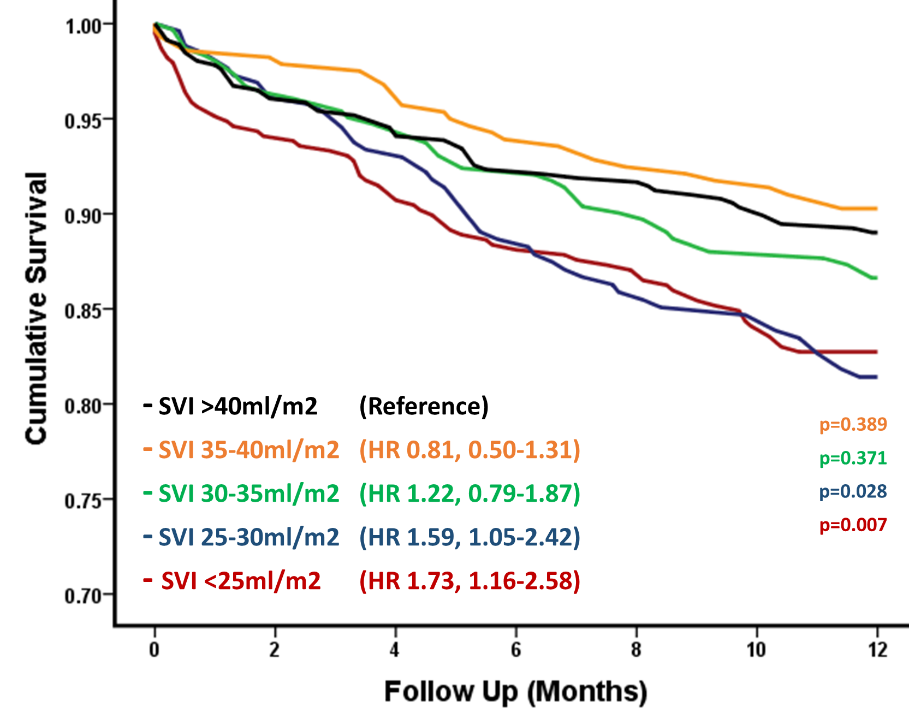


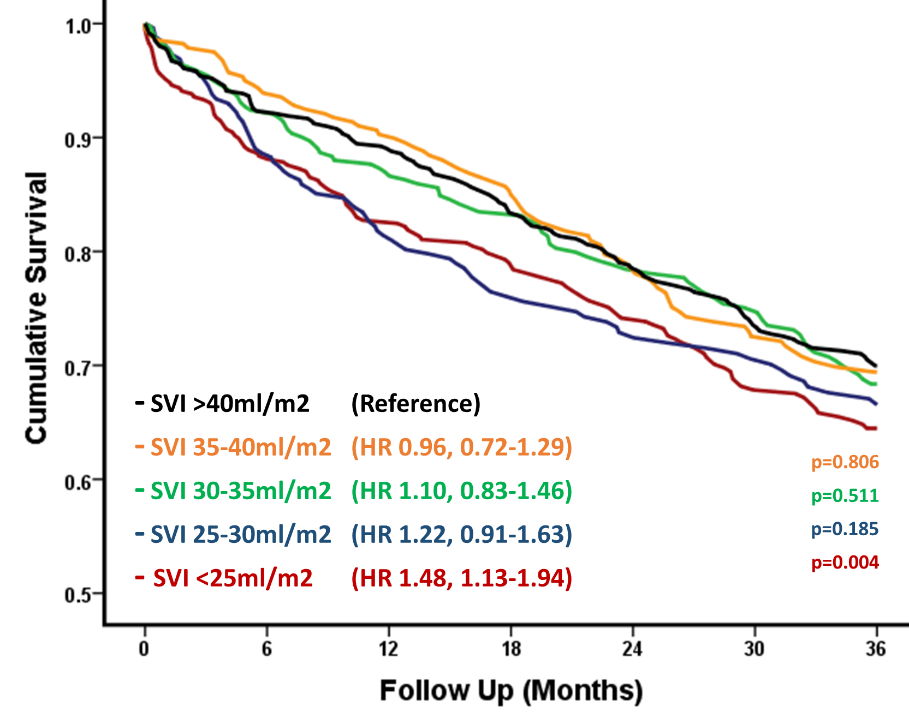


Table S2: Baseline Group Characteristics According to SVI subgroup **only including** **female** **patients** with low-gradient severe AS and preserved LVEF (≥50%)

| **Variable** | **SVI <25ml/m^2^ n=273** | **SVI 25-30ml/m^2^ n=153** | **SVI 30-35ml/m2 n=176** | **SVI >35ml/m^2^ n=470** |
| --- | --- | --- | --- | --- |
| BMI | 29.2 (±7.8)** | 27.6 (±6.3) | 28.0 (±7.5)* | 26.3 (±5.8) |
| LVOT Diameter (cm) | 1.66 (±0.25)** | 1.78 (±0.21)** | 1.87 (±0.21)** | 2.01 (±0.19) |
| AVA (VTI) (cm^2^) | 0.84 (±0.24)** | 0.85 (±0.22)** | 0.87 (±0.21)* | 0.93 (±0.16) |
| Index AVA (VTI) (cm^2^/m^2^) | 0.47 (±0.14)** | 0.50 (±0.14)** | 0.51 (±0.12)** | 0.56 (±0.11) |
| AV Mean Gradient (mmHg) | 11.9 (±8.5)** | 17.7 (±8.3)** | 21.5 (±8.1)** | 26.6 (±7.0) |
| AV Peak Velocity (m/s) | 2.2 (±0.8)** | 2.7 (±0.6)** | 3.0 (±0.6)** | 3.4 (±0.4) |
| LVEF (%) | 63.6 (±8.3) | 63.5 (±6.5) | 64.5 (±7.7) | 64.7 (±7.7) |
| LVEDD (cm) | 4.2 (±0.6) | 4.0 (±0.6) | 4.1 (±0.6) | 4.1 (±0.6) |
| LVESD (cm) | 2.7 (±0.5)* | 2.6 (±0.5) | 2.6 (±0.5) | 2.6 (±0.5) |
| RVSP (mmHg) | 41.8 (±16.6) | 41.9 (±13.8) | 41.4 (±13.4) | 41.6 (±14.0) |
| Cardiac Damage Stage |  |  |  |  |
| Stage 0 | 35.9% (98)* | 32.7% (50) | 28.4% (50) | 26.4% (124) |
| Stage 1 | 13.6% (37) | 20.9% (32) | 19.9% (35) | 15.5% (73) |
| Stage 2 | 25.6% (70)* | 17.0% (26)** | 30.1% (53) | 39.1% (184) |
| Stage 3/4 | 24.9% (68) | 29.4% (45)* | 21.6% (38) | 18.9% (89) |
| AVR during follow-up⸶ | 5.1% (14)** | 7.2% (11)** | 13.6% (24) | 20.0% (94) |
| Mean follow-up (months) | 58.4 (±34.8)** | 67.4 (±40.6)* | 71.2 (±44.4) | 81.0 (±43.6) |

Figure S3

Showing one- and three-year Kaplan-Meier survival curves in **only female patients** with severe low-gradient AS and preserved EF (≥50%) according to SVI subgroup, including results of Cox proportional hazards multivariable regression analyses adjusting for patient age, sex, BMI, indexed AVA and Cardiac Damage Stage.


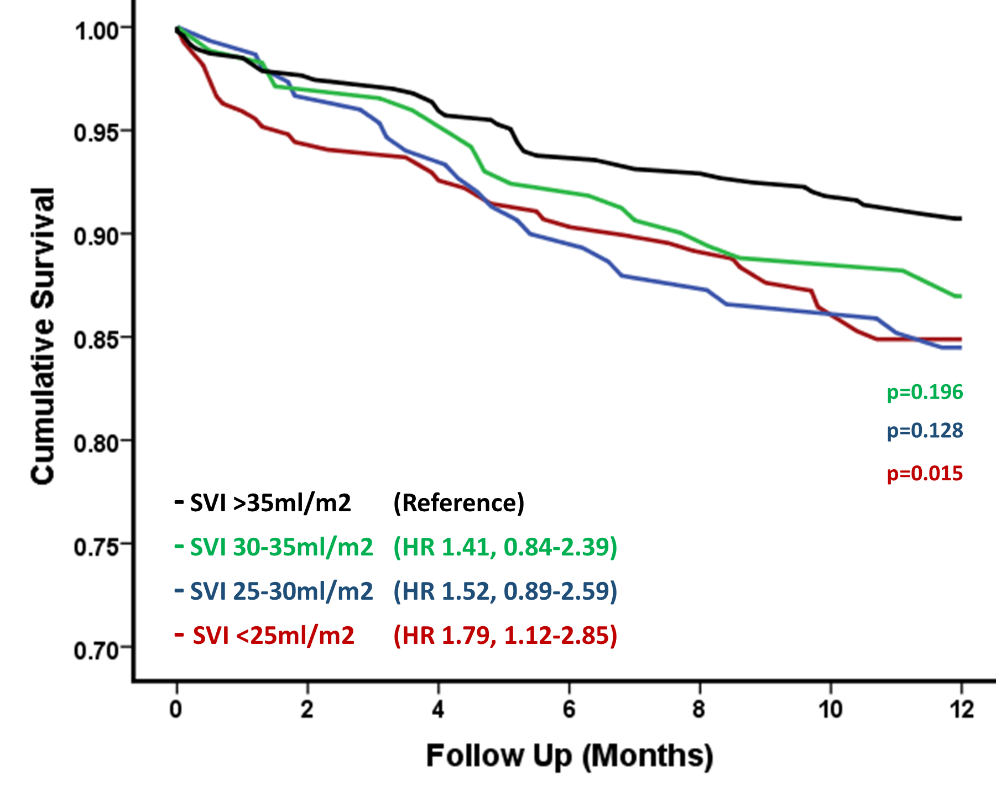


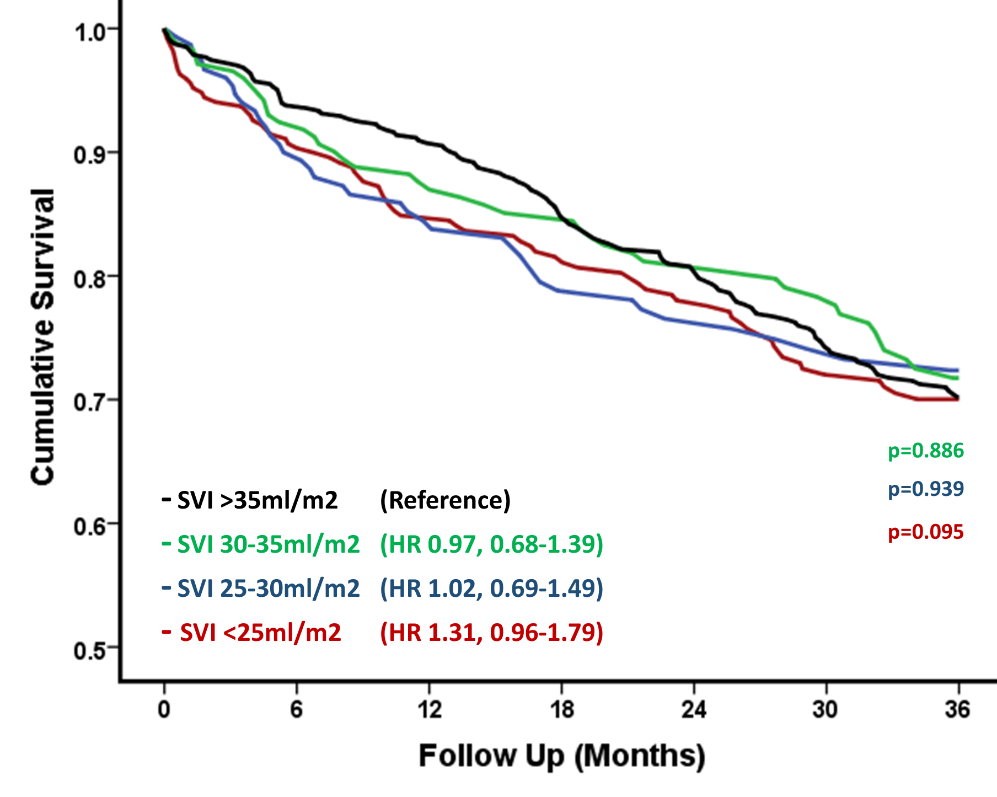


Table S3:

Baseline Group Characteristics According to SVI subgroup **only including male patients** with low-gradient severe AS and preserved LVEF (≥50%)

| **Variable** | **SVI <25ml/m^2^ n=119** | **SVI 25-30ml/m^2^ n=108** | **SVI 30-35ml/m2 n=130** | **SVI >35ml/m^2^ n=270** |
| --- | --- | --- | --- | --- |
| BMI | 29.1 (±6.4)** | 28.8 (±6.2)** | 28.6 (±5.0)** | 26.1 (±4.5) |
| LVOT Diameter (cm) | 1.87 (±0.27)** | 2.04 (±0.24)** | 2.05 (±0.24)** | 2.18 (±0.21) |
| AVA (VTI) (cm^2^) | 0.83 (±0.31)** | 0.92 (±0.35) | 0.92 (±0.27) | 0.95 (±0.17) |
| Index AVA (VTI) (cm^2^/m^2^) | 0.42 (±0.13)** | 0.47 (±0.19)* | 0.48 (±0.17)* | 0.52 (±0.13) |
| AV Mean Gradient (mmHg) | 15.4 (±8.3)** | 21.6 (±9.3)** | 25.6 (±7.7)* | 28.8 (±7.1) |
| AV Peak Velocity (m/s) | 2.5 (±0.6)** | 3.0 (±0.6)** | 3.3 (±0.5)** | 3.5 (±0.4) |
| LVEF (%) | 60.7 (±7.5) | 61.6 (±8.5) | 63.0 (±8.0) | 62.1 (±7.1) |
| LVEDD (cm) | 4.5 (±0.7) | 4.5 (±0.6) | 4.6 (±0.5) | 4.5 (±0.6) |
| LVESD (cm) | 3.0 (±0.6) | 2.9 (±0.6) | 2.9 (±0.6) | 3.0 (±0.6) |
| RVSP (mmHg) | 43.3 (±16.2)* | 42.3 (±12.6) | 41.0 (±12.5) | 38.3 (±10.8) |
| Cardiac Damage Stage |  |  |  |  |
| Stage 0 | 39.5% (47) | 34.3% (37) | 35.4% (46) | 33.3% (90) |
| Stage 1 | 8.4% (10) | 13.9% (15) | 24.6% (32) | 16.3% (44) |
| Stage 2 | 28.6% (34) | 37.0% (40) | 25.4% (33)* | 42.2% (114) |
| Stage 3/4 | 23.5% (28)** | 14.8% (16) | 14.6% (19) | 8.1% (22) |
| AVR during follow-up⸶ | 11.8% (14)** | 14.8% (16)* | 23.1% (30) | 33.3% (90) |
| Mean follow-up (months) | 65.0 (±31.1)* | 64.6 (±40.4)* | 78.4 (±40.4) | 80.2 (±44.9) |

Figure S4

Showing one- and three-year Kaplan-Meier survival curves in **only male patients** with severe low-gradient AS and preserved EF (≥50%) according to SVI subgroup, including results of Cox proportional hazards multivariable regression analyses adjusting for patient age, sex, BMI, indexed AVA and Cardiac Damage Stage.


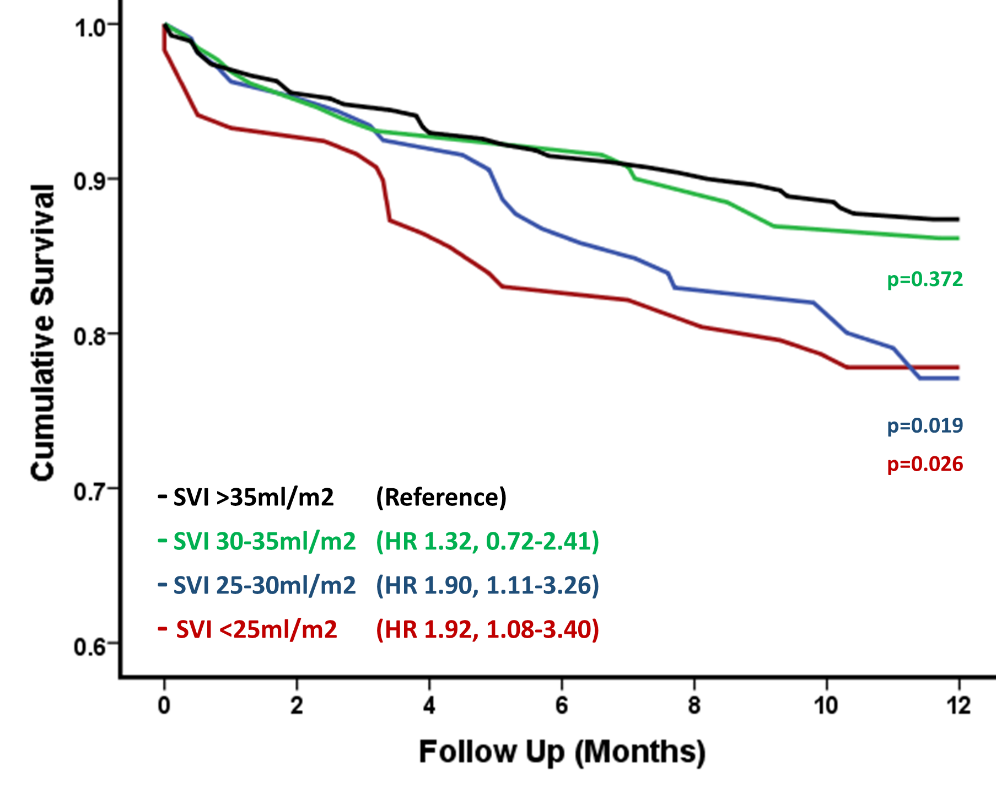


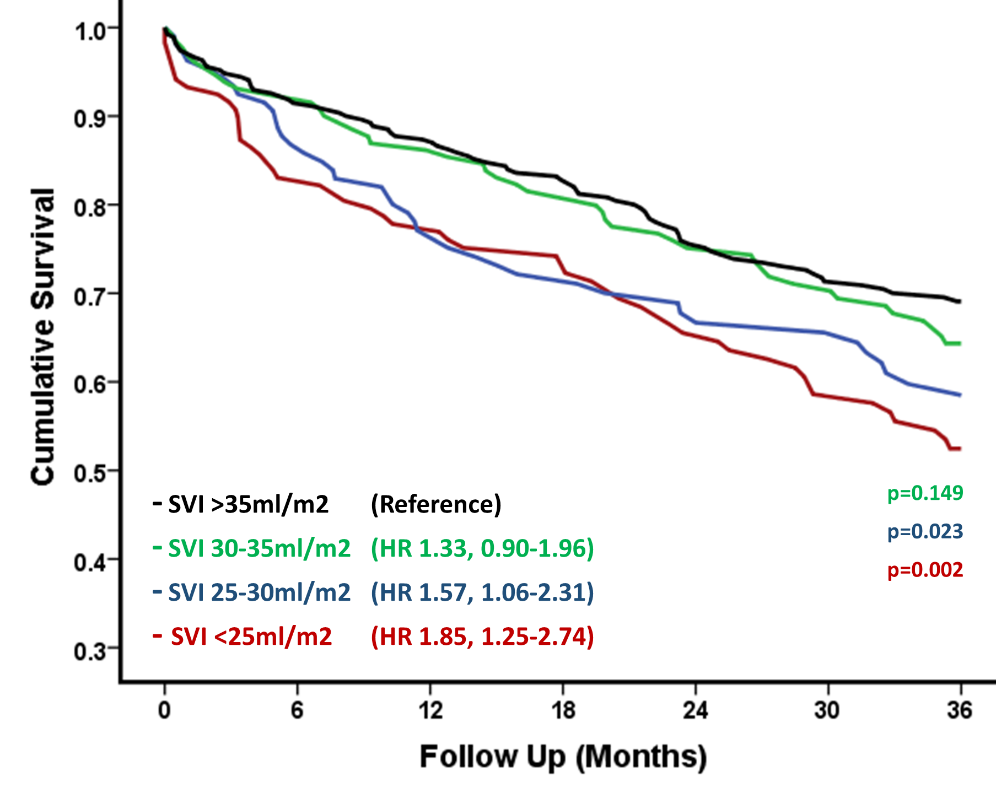

Supplement: Supplementary file 1 — Supplementary file1 (DOCX 1042 KB) [file 10554_2023_2886_MOESM1_ESM.docx]
